# Exacerbation of Psychogenic Non-epileptic Seizures Related to the Diagnosis and Disease Burden of Epilepsy: A Case Report

**DOI:** 10.7759/cureus.68445

**Published:** 2024-09-02

**Authors:** Kenichi Shizukawa, Yuichi Nakamura, Kenki Yokoyama, Yutaka Fujii, Toru Horinouchi

**Affiliations:** 1 Department of Psychiatry and Neurology, Hokkaido University Hospital, Sapporo, JPN; 2 Department of Psychiatry, Hokkaido Prefectural Koyogaoka Hospital, Abashiri, JPN; 3 Department of Psychiatry, Iwamizawa Municipal General Hospital, Iwamizawa, JPN

**Keywords:** comorbid conditions, conversion disorder, disease burden, epilepsy, epileptic seizures, psychoeducation, psychogenic non-epileptic seizures, psychological distress, social stigma

## Abstract

Psychogenic non-epileptic seizures (PNES), which closely resemble epileptic seizures (ES), are typically triggered by psychological distress and represent the most prevalent form of conversion disorder encountered in clinical practice. Multiple physical conditions can both precipitate and sustain PNES episodes. Epilepsy, a common neurological disorder, imposes significant emotional and physical burdens, frequently resulting in elevated levels of anxiety and depression. This case report details the clinical course of a 19-year-old female whose PNES was exacerbated by the diagnosis and disease burden of epilepsy. The patient's background of childhood trauma, bullying, and sexual abuse likely predisposed her to the development of PNES. Upon receiving a diagnosis of epilepsy, characterized by focal seizures originating from the left parietal region, the patient experienced increased anxiety and required frequent hospitalizations. Despite adjustments to her treatment regimen, including the administration of levetiracetam (LEV) and lacosamide (LCM), her seizures persisted. Comprehensive evaluations, comprising electroencephalography (EEG) and single-photon emission computed tomography (SPECT), indicated the coexistence of epilepsy and PNES. Although surgical intervention was initially considered, it was ultimately deemed unnecessary, which subsequently alleviated the patient’s anxiety. Psychoeducation highlighting the manageability of her epilepsy with ongoing pharmacotherapy significantly reduced her PNES episodes. This case emphasizes the critical role of addressing the psychosocial burden associated with an epilepsy diagnosis, as these factors may exacerbate PNES. It also underscores the importance of a holistic treatment approach that integrates psychological support with medical management.

## Introduction

Psychogenic non-epileptic seizures (PNES) manifest as episodes characterized by abnormal motor, sensory, and/or cognitive changes that closely mimic epileptic seizures (ES) [1]. These episodes are considered unconscious and involuntary expressions of psychological distress, comparable to panic attacks [2]. Psychogenic non-epileptic seizures are recognized as the most prevalent form of conversion disorder, with the term PNES widely utilized in contemporary clinical practice [1,2].

The current literature underscores the significant coexistence of various physical disorders with PNES [3]. For instance, previous studies have identified that comorbid conditions such as migraines, asthma, hypertension, diabetes, and thyroid disorders can act as both precipitating and perpetuating factors for PNES [3,4]. As a result, the identification and management of these comorbidities are crucial in developing individualized treatment plans for PNES patients [4].

Epilepsy, a prevalent neurological disorder, affects individuals of all ages and socioeconomic backgrounds globally, imposing a considerable disease burden [5]. Emotional distress, including anxiety and depression, is frequently observed among individuals with epilepsy and can significantly impair their quality of life [6]. This suggests that epilepsy itself may serve as a precipitating and perpetuating factor for PNES.

However, there is a notable lack of research specifically examining how the process of epilepsy diagnosis and the associated psychological burden contribute to the onset or exacerbation of PNES. Thus, this case report describes a unique instance where PNES was exacerbated following an epilepsy diagnosis and the ensuing disease burden. The objective of this report is to elucidate the interplay between epilepsy diagnosis, disease burden, and the exacerbation of PNES.

## Case presentation

The patient was a 19-year-old right-handed Japanese female who was born at full term without complications. At the age of five, following an incident of sexual abuse involving a family member, her parents divorced, and she was placed in the custody of her mother. At the age of 13, the patient began experiencing seizure episodes characterized by eye closure, falling, and head shaking, which were triggered by bullying at junior high school. She sought care at a local psychiatric hospital, where a comprehensive evaluation, including blood tests, an electrocardiogram, brain magnetic resonance imaging (MRI), and electroencephalography (EEG), revealed no abnormalities, leading to a diagnosis of PNES. The seizures subsided after the patient was transferred to a special support class and began attending biweekly psychotherapy sessions. At the age of 14 years, the patient was assessed using the Wechsler Intelligence Scale for Children-Fourth Edition. She scored a Full Scale Intelligence Quotient (FSIQ) score of 89, thereby ruling out intellectual disability.

In January 2023, the patient experienced a series of tonic-clonic seizures, which were preceded by prodromal symptoms, including ascending abdominal discomfort, olfactory hallucinations, and impaired consciousness. She was subsequently transported to a local emergency hospital, where the seizures were alleviated following the intravenous administration of diazepam. Levetiracetam (LEV) 1000 mg was prescribed, resulting in further seizure control. In February 2023, for a definitive diagnosis and comprehensive evaluation of epilepsy, she was referred to the Department of Psychiatry and Neurology at our institution, where she had her initial consultation.

**Figure 1:**
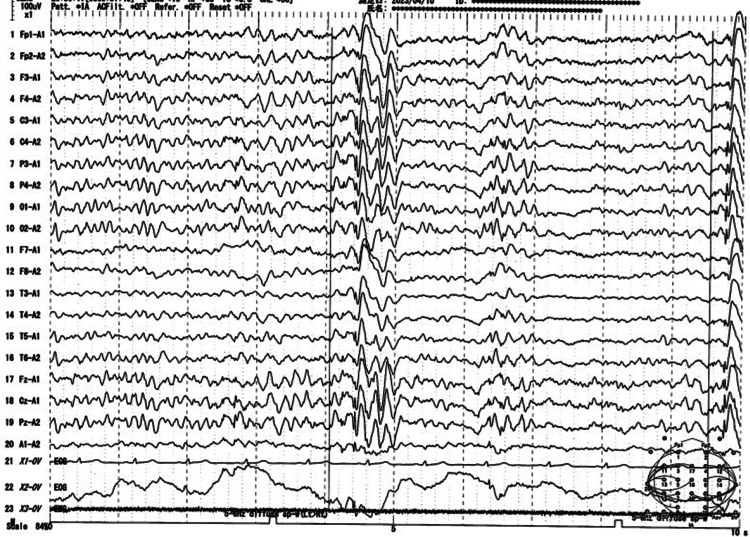
Continuous electroencephalogram A 3-5 Hz, 150-200 μV generalized spike-and-slow-wave complex originating in the left parietal region

Following this consultation, a comprehensive evaluation, including laboratory workup and brain MRI, was conducted to rule out symptomatic epilepsy. An EEG was performed to detect any abnormal activity suggestive of generalized or focal epilepsy. The EEG revealed a spike-and-slow-wave complex that originated focally in the left parietal region and subsequently became generalized (Figure 1). Consequently, we concluded that the patient had focal epilepsy with a seizure focus in the left parietal region. Since her seizures were already well-controlled, we opted to continue outpatient care with LEV 1000 mg and provided education about epilepsy.

At the follow-up visit in March 2023, we explained the diagnosis of epilepsy based on the observed EEG abnormalities and advised her to seek emergency assistance in the event of prolonged seizures. The patient expressed significant distress, stating, "I feel like another burden has been added." Subsequently, she experienced four emergency transports and three hospital admissions due to recurrent seizures. The dosage of LEV was titrated up to 3000 mg, but it failed to demonstrate efficacy. The treatment regimen was modified to lacosamide (LCM) at a dosage of 400 mg. Despite this adjustment, there was no observed improvement in the frequency or duration of the seizures. Subsequently, adequate doses and sufficient duration of carbamazepine and phenytoin were administered sequentially, but these interventions also failed to achieve adequate seizure control.

**Figure 2:**
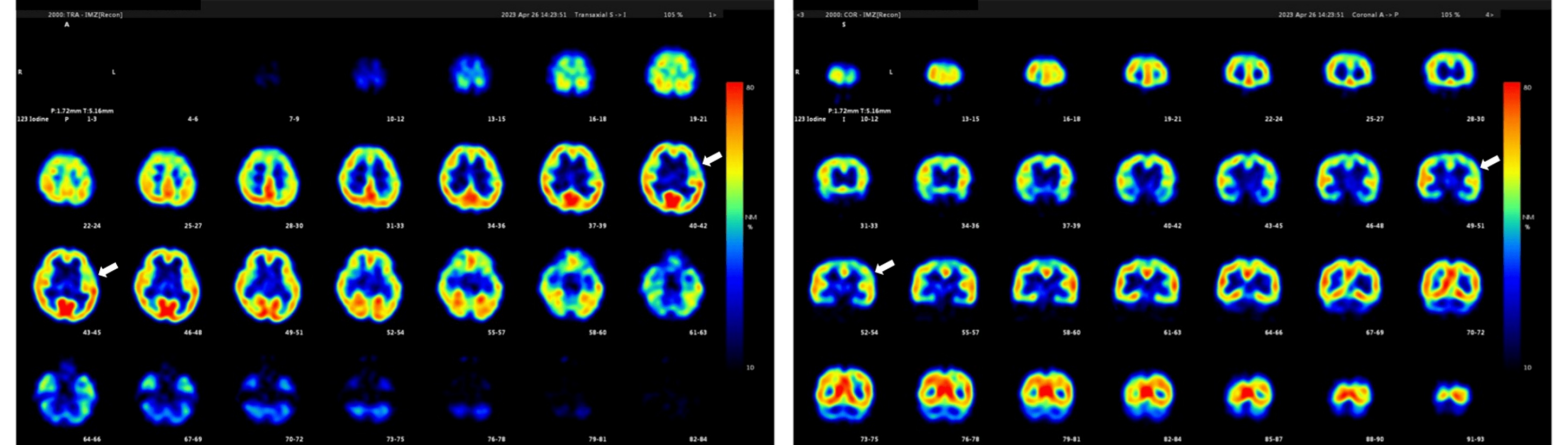
The 123I-IMZ-SPECT reports of the patient Decreased accumulation around the left insular cortex during the interictal period (white arrows) 123I-IMZ-SPECT: iodine-123-iomazenil single-photon emission computed tomography

In April 2023, we decided to conduct a comprehensive epilepsy evaluation. The iodine-123-iomazenil single-photon emission computed tomography (123I-IMZ-SPECT) demonstrated decreased tracer uptake in the left insular cortex during the interictal phase, suggesting potential cortical neuron damage or a loss of neuronal integrity in the affected region (Figure 2). During long-term video EEG (LT-VEEG) monitoring, five seizure episodes lasting approximately 10 minutes each were documented. Each episode was characterized by the sudden onset of epigastric discomfort and olfactory hallucinations, followed by tonic rigidity and clonic convulsions of the limbs. Excessive drooling, urinary incontinence, sinus tachycardia, and impaired consciousness were documented. Despite the presence of these clinical manifestations, the EEG did not reveal an epileptic focus, aside from muscle and motion artifacts. Despite the absence of definitive EEG abnormalities, the presence of prodromal visceral-sensory symptoms, including epigastric discomfort and olfactory hallucinations, along with cortical neuronal abnormalities identified in the left insular lobe on 123I-IMZ-SPECT, strongly suggested the presence of insular lobe epilepsy. This hypothesis was further supported by the well-documented difficulty in capturing insular lobe seizures on surface EEG due to the deep anatomical location of the insular cortex. Consequently, we planned to collaborate with the epilepsy surgery team and conduct a psychological assessment.

In May 2023, we informed the patient that her recent episodes were diagnosed as ES rather than PNES, although we acknowledged that some episodes could still be PNES. We further explained that based on EEG and 123I-IMZ-SPECT findings, insular lobe epilepsy was the most likely diagnosis. Given that insular lobe epilepsy is often refractory to pharmacotherapy, and considering the patient's lack of response to a regimen of four antiseizure medications, a diagnosis of drug-resistant epilepsy was made. Therefore, we discussed the potential benefits of surgical intervention and recommended a referral to the epilepsy surgery center at another university hospital for a comprehensive evaluation. Despite these discussions, the patient expressed significant confusion, stating, "My mind is in chaos." Accompanied by her mother, the patient proceeded to the epilepsy surgery center as recommended, where a diagnostic admission was planned to assess the indication for surgical treatment. 

While awaiting diagnostic admission, the patient experienced an exacerbation in seizure frequency, accompanied by episodes of limb paralysis, resulting in recurrent falls and injuries. This clinical deterioration necessitated inpatient management in the psychiatric unit. Following her admission, the counseling service she had relied on became unavailable due to its unexpected closure. Furthermore, her high school administration notified us that continued enrollment would be difficult due to the refractory nature of her epilepsy. Subsequently, the patient exhibited increased anxiety and irritability, along with impulsive behaviors such as peeling off protective coverings from the corners of her hospital room used to prevent injuries. Seizure frequency further escalated, with the patient experiencing two to three seizures daily, often without any preceding aura. These seizures predominantly occurred during the day, each lasting more than 20 minutes, and were characterized by eye closure, unresponsiveness, and weeping. Given these clinical manifestations, the likelihood of PNES was considered high. Crisis intervention involving multiple attending physicians and regular counseling sessions conducted by qualified psychiatric nurses were implemented to alleviate her psychological burden. However, there was no observed improvement in the frequency or duration of the seizures.

In November 2023, a diagnostic admission at the epilepsy surgery center revealed no EEG abnormalities during LT-VEEG monitoring. Six seizures, characterized by tonic-clonic movements of both upper and lower limbs, were recorded; however, all episodes were ameliorated with the administration of oxygen and haloperidol. Despite the seizure duration exceeding 40 minutes, no EEG abnormalities, decreases in oxygen saturation, or postictal confusion were observed, suggesting the presence of PNES. Additionally, while the patient did not exhibit symptoms such as eye closure, upward eyeball deviation, or falls prior to May 2023, these symptoms began to appear from June 2023 onwards. The fluctuation in seizure presentation further indicated the presence of PNES. Consequently, it was determined that the seizures observed during the diagnostic admission were PNES, leading to the conclusion that the patient was not a candidate for surgical intervention.

In response to the complex condition, we implemented a multifaceted treatment strategy encompassing both pharmacological and psychosocial interventions. Lacosamide was prescribed at a dosage of 400 mg daily as part of the antiepileptic and psychotropic medication regimen. To ensure rigorous monitoring and adaptability of the treatment plan, biweekly outpatient visits were scheduled, allowing for continuous assessment and adjustment of the treatment as necessary. In conjunction with these visits, she participated in biweekly psychotherapy sessions with a clinical psychologist at a local psychiatric hospital, aimed at providing sustained psychological support. A critical component of management involved comprehensive psychoeducation on epilepsy and PNES extended to both the patient and her support network. These sessions clarified that her recent seizures were attributed to PNES and reinforced that her epilepsy was not refractory and could be effectively managed through ongoing pharmacotherapy. We directly addressed the patient’s heightened anxiety and fear related to the perceived refractory nature of her epilepsy, which had contributed to the exacerbation of PNES symptoms. The therapeutic focus was placed on encouraging the patient to maintain her daily life with minimal concern over her condition. To further enhance her support system, targeted psychoeducation regarding epilepsy and PNES, including seizure management strategies, was provided to the staff in her high school. Her response to these interventions was notably positive; she expressed a sense of relief, stating, "I can live as I did before. I feel so relieved." Consequently, the frequency of her PNES episodes significantly decreased to approximately 0 to three times per month.

## Discussion

Several studies have explored the relationship between epilepsy and PNES. The prevalence of PNES among patients with epilepsy has been reported to be approximately 12%, suggesting that epilepsy is associated with a higher incidence of PNES [7]. The use of antiseizure medications can also aggravate dissociative states, potentially facilitating the development of PNES [8]. In individuals diagnosed with both epilepsy and PNES, PNES almost always follows the onset of epileptic seizures [9,10], with instances of PNES occurring before epilepsy being extremely rare. This case report is the first to document a recurrence of previously remitted PNES following a new epilepsy diagnosis, suggesting that PNES can precede ES. This case highlights that the disease burden of epilepsy may play a critical role in the onset or exacerbation of PNES.

While the connection between epilepsy and PNES is well-established, it is equally important to consider the impact of past trauma, particularly sexual abuse, on the development and progression of PNES. Research has shown that a history of sexual abuse is significantly associated with an increased risk of developing PNES [11]. Moreover, patients with PNES who have experienced sexual abuse often exhibit more severe dissociative and depressive symptoms compared to those without such a history [12]. These findings highlight that traumatic childhood experiences, such as sexual abuse, can create a heightened vulnerability to PNES, particularly in individuals who later develop epilepsy. The frequent coexistence of epileptic and nonepileptic seizures in these patients highlights the necessity of a comprehensive treatment strategy that addresses both the neurological and psychological dimensions of their condition.

Epilepsy is associated with increased anxiety, depressive symptoms, and a decrease in self-esteem [13]. The unpredictable and uncontrollable nature of ES can induce learned helplessness and diminish self-efficacy. Furthermore, a lack of social resources, a fragile support network, and financial hardship can exacerbate anxiety, depressive symptoms, and reduced self-efficacy [14]. In Japan, a diagnosis of epilepsy often results in significant social stigma, leading to disadvantages in education, employment, and marriage [15].

Stigma plays a profound role in the psychological and social challenges faced by individuals with epilepsy. The societal perception of epilepsy as a disorder with severe and uncontrollable symptoms can lead to social isolation and discrimination. In this case, her experience of stigma was compounded by her high school administration's decision that she could no longer attend classes due to her condition. This institutional response not only reinforced her feelings of inadequacy but also disrupted her routine and support system, further exacerbating PNES.

The inconsistency of psychiatric support systems also contributed to her deteriorating condition. The closure of the counseling service she had relied on, coupled with the lack of a stable support network during a critical phase of her illness, left her vulnerable to the exacerbation of PNES. This case underscores the critical need for consistent and accessible psychiatric care, particularly for patients who are managing the dual burden of epilepsy and PNES.

Emphasizing the possibility of refractory epilepsy and the need for surgery further heightened her anxiety and exacerbated PNES. When treating newly diagnosed epilepsy, it is essential to consider the potential psychosocial burden of the diagnosis and treatment, as these factors may contribute to the development or exacerbation of PNES.

## Conclusions

This case illustrates the complex interplay between epilepsy and PNES, where the diagnosis and associated disease burden of epilepsy appeared to exacerbate the patient’s PNES. The patient’s history of childhood and adolescent trauma, including bullying and sexual abuse, likely predisposed her to PNES. However, it was the patient's profound anxiety and fear regarding her epilepsy diagnosis that played a pivotal role in the recurrence of PNES. The cumulative physical, financial, and emotional stressors associated with epilepsy overwhelmed her coping mechanisms, leading to a significant exacerbation of PNES symptoms. This case underscores the importance of comprehensive psychoeducation that not only addresses the medical aspects of epilepsy but also specifically targets psychological interventions to mitigate excessive worry and prevent the exacerbation of PNES. Therefore, when providing psychoeducation or making environmental adjustments for patients newly diagnosed with epilepsy, clinicians must be vigilant about the potential of the diagnosis and disease burden to trigger or worsen PNES.
